# Could belimumab be a new weapon in autoimmune complicated CVID?

**DOI:** 10.3389/fimmu.2026.1805917

**Published:** 2026-05-14

**Authors:** Cristiano Maiolo, Paola Lucia Minciullo, Giuseppe Murdaca, Sebastiano Gangemi

**Affiliations:** 1 Department of Clinical and Experimental Medicine, School and Operative Unit of Allergy and Clinical Immunology, University of Messina, Messina, Italy; 2 Department of Internal Medicine, University of Genova, Genova, Italy; 3 Allergology and Clinical Immunology Unit, San Bartolomeo Hospital, Sarzana, Italy

**Keywords:** BAFF, belimumab, CD21^Low^, CVID, lupus, T-bet^Hi^

## Introduction

CVID (Common Variable Immunodeficiency) is a heterogeneous disorder that represents the most frequent symptomatic primary immunodeficiency (PID, sometimes referred to as Primary Immune Dysregulation Disorders or PIDD), defined by hypogammaglobulinemia, impaired vaccine responses, frequent and/or severe infections, but also characterized by symptoms related to autoimmunity and autoinflammation, such as cytopenias, granulomatous disease, and unexplained polyclonal lymphoproliferation (1). Despite the name suggesting a condition characterized by frequent and severe infections, the growing body of epidemiological and clinical data has led to a more precise subdivision of patients according to their main clinical manifestations, as agreed in the 2016 ICON consensus (2):

- Autoimmune/Inflammatory, which occurs approximately in 25-30% of CVID patients (cytopenia mainly)
- Enteropathy (unexplained—excluding infective, autoimmune, and glutensensitive enteropathies) which affects approximately 9% of patients
- No disease-related complications (described as ‘‘infections only’’)
- Lymphoproliferation (including persistent unexplained lymphadenopathy and splenomegaly) in almost 20% of patients
- Interstitial Lung Disease (2, 3)

Furthermore, in 2008, the EUROClass trial established a new classification tool to define CVID patient risk profiles by accounting for three parameters: total B cells, CD27^+^IgD^-^ B cells (switched memory), CD21^Low^ B cells (4).

The trial had provided new insights into the pathogenesis of CVID by linking cellular immunophenotyping to clinical phenotypes. Specifically, autoimmune and inflammatory complicated CVID (CVID-AI) constitute phenotypes with a possible shared pathogenetic substrate as multiple studies showed that the aforementioned non-infectious manifestations were related to increased levels of CD21^Low^ B cells (4, 5).

Management of CVID-AI patients is often challenging and risky due to their susceptibility to infections. Nonetheless, these patients frequently receive non-specific immunosuppressive agents, with the risk of further damaging an already impaired immune system (6). In current medical practice the management of autoimmune and granulomatous related complications of CVID is firmly anchored to steroids use and, in resistant cases of cytopenias, to splenectomy (2). Rituximab, an anti-CD20 chimeric monoclonal antibody, has gained a pivotal role in maintaining remission in both autoimmune and ILD complications (7–9); however, anti-CD20 therapy should still be considered a broad-spectrum immunosuppressant associated with a significant risk of infections.

In this context it’s relevant to highlight how new research and manufacturing technologies are driving health sciences toward the frontier of precision medicine, where the ability to identify and target specific molecular pathways (cytokines, receptors, protein kinases) is, and increasingly will be, capable of achieving more effective clinical outcomes with fewer adverse effects.

Monoclonal antibodies have radically transformed the management of immune mediated diseases resulting in substantial clinical improvements while markedly reducing long-term systemic corticosteroid use and its associated adverse effects. This is clearly evident in the field of Th2 axis, where monoclonal antibodies targeting cytokines or molecules (IL-4/13, IL-5, TSLP and IgE) have shown that, despite concerns expressed by part of the scientific community, these drugs have a solid and reliable safety profile as demonstrated in related scientific literature (10).

In recent years, belimumab, a monoclonal antibody targeting BAFF (B cell activating factor), has changed the therapeutic approach to Systemic Lupus Erythematosus (SLE), resulting in a marked reduction in the use of systemic corticosteroids and conventional DMARDs, with a significant impact on disease activity as measured by SLEDAI, an index activity score consisting of both clinical and serological parameters (e.g. aphthosis, fever, arthritis, headache, leukopenia, c3 and c4 reduction, or elevated n-DNA titer, 11, 12). Furthermore belimumab proved effective in reducing the ESSDAI score, main activity index for Sjogren Syndrome (SS) consisting of 12 clinical, biological and immunological domains (13), both in monotherapy (14) and combined therapy with Rituximab (15).

BAFF is a ligand of the TNF family, produced by monocyte-derived dendritic cells and T cells, whose main receptor (BAFF-R/BR3) has been shown to be expressed almost exclusively on B cells, nonetheless BAFF exerts its biological effects through three different receptors: BR3, calcium-modulating cyclophilin ligand interactor (TACI) and BCMA. Through BR3, BAFF promotes the survival of CD38^+^ activated memory B cells and seems to be involved in autoimmunity via plasmablast survival and activation (16).

**Figure 1:**
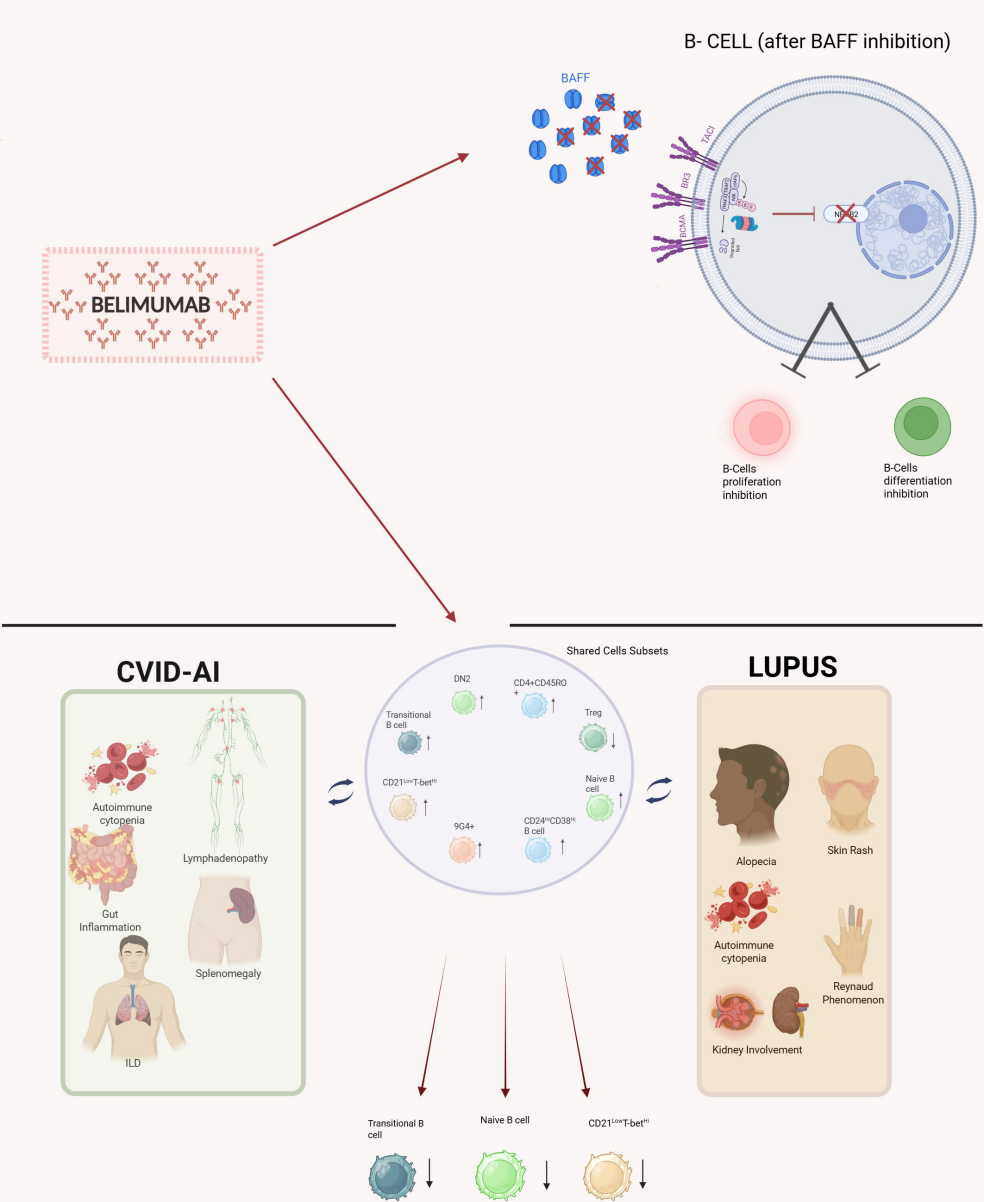
Schematic overview of: schematic representation of belimumab targeting BAFF and inhibiting B-cell differentiation and proliferation via NF-kB2 inhibition; clinical manifestation of SLE, CVID-AI, their shared cellular compartments and B cells subsets reduced by belimumab. Created in BioRender. Maiolo, C. (2026) https://BioRender.com/ak7urpn. BAFF, B-Cell Activating Factor; BCMA, B-Cell Maturation Antigen; BR3, BAFF-receptor; CVID-AI, Common Variable Immunodeficiency with Autoimmune or Inflammatory Manifestations; DN2, Double Negative 2 B cells; ILD, Interstitial Lung Disease; Nf-kB2, Nuclear factor kappa-light-chain-enhancer of activated B cells; NIK, NF-κB-inducing kinase; SLE, Systemic Lupus Erytehmatosus; TACI, Calcium-modulating Cyclophilin Ligand Interactor; TRAF, TNF- Receptor Associated Factors; Treg, Regulatory T cells.

Belimumab exerts a selective action by targeting only BAFF-dependent B-cell subsets, without depleting switched memory B cells or plasma cells (15, 17, 18). Considering its biological targets (**Figure 1**) and the available literature, which reports very rare adverse events (19), belimumab could be regarded as an immune modulator rather than an immunosuppressive agent.

Despite the absence of any published data or experience with belimumab in CVID-AI, and given its safety and efficacy profile in SLE and SS patients, as well as the similarity of some clinical manifestations of SLE and SS with CVID-AI, we began investigating pathogenetic similarities in order to discuss a hypothesis-driven, targeted strategy for selected inflammatory phenotypes characterized by presumed BAFF-mediated B-cell dysregulation.

## CVID-AI, SLE and SS, what do they have in common?

Although CVID is generally considered a B cell–driven disease, it is not uncommon to find alterations in T-cell number and activation, as well as abnormalities affecting dendritic cells or certain cytokines (20). Therefore, many studies have investigated over the years how B-cell, T-cells subsets and cytokines may be involved in CVID pathogenesis, defining a clearer picture of immune system alterations in these patients.

As expected, CVID-AI patients display not only an increased proportion in CD21^Low^ B cells but also a reduced proportion of regulatory T cells, including both CD3^+^CD25^+^CD127^Low^ and CD4^+^CD25^+^FoxP3^+^ Tregs, compared with healthy controls (5). An expansion of CD4^+^CD45RO^+^ T cells, believed to represent a senescent subset (21), has also been observed, with their frequency negatively correlating with Treg levels (5).

CD21^Low^ B cells, considered a hallmark of chronic adaptive immune system activation (22), appear to play a central role in CVID immune dysregulation. In a cohort of 72 CVID patients, 69% exhibited increased CD21^Low^ B cells, while 38.5% showed a Treg defect, with a clear negative correlation between these two populations. Notably, autoimmune features were present in 80% of patients with reduced Treg levels (23). A similar immunological pattern with lower Tregs (24) and higher CD4+CD45RO+ Th cells has been described in SLE, where reduced Treg numbers and function correlated with disease activity as measured by SLEDAI (21) and were associated with decreased Interleukin 2 (IL-2) levels (24, 25). Consistently, IL-2 deficiency has also been reported in CVID, where cytokine supplementation resulted in increased antibody production (26).

In SLE, the CD21^Low^ population was significantly expanded compared to healthy donors and to other autoimmune diseases in which CD21^Low^ expansion was less relevant (27, 28). Notably, CD21^Low^ levels were particularly expanded in patients with active disease, correlating with higher SLEDAI scores (29).

BAFF (B-cell activating factor), also known as BlyS, seems to be of main importance in CVID-AI and connective tissue diseases. In murine models, increased BAFF levels have been associated with splenomegaly, autoimmunity, B-cell hyperplasia, and hypergammaglobulinemia. BAFF was found to be increased in CVID patients, in whom *in vitro* studies demonstrated that B cells proliferate and enhance IgM production after exposure to BAFF. Notably, studies on autoimmunity have shown that autoantigen-engaged B cells have increased dependence on BAFF and can be rescued from elimination by high BAFF levels (20). Furthermore, higher BAFF levels have been related to Interstitial Lung Disease (ILD) presentation, where the interplay between BlyS and naïve B cells leads to B-cell hyperplasia, probably relevant in CVID-ILD pathogenesis.

CVID-ILD affects up to one out of three CVID patients and may manifest within the spectrum of benign lymphoproliferative lung pathology, ranging from follicular bronchiolitis to nodular lymphoid hyperplasia with granulomatous inflammation, which is often a feature of this form of ILD. Moreover, in CVID, ILD is frequently associated with cytopenias and splenomegaly, suggesting a shared pathogenetic driver. Interestingly, progressive ILD in CVID has been found to be associated with increased IgM levels, again suggesting an important role for BAFF in CVID-ILD pathogenesis (30).

Moreover, secondary manifestations of CVID, such as cytopenias, enteropathy, interstitial lung disease, and lymphadenopathy, have been shown to correlate positively with IFN-γ levels. In these patients, CD21^Low^ B cells have been characterized as T-bet^Hi^, a subset whose proliferation appears to be T cell–dependent, driven by both T follicular helper and peripheral helper cells, and associated with an interferon signature (22). An accumulation of CD21^Low^ T-bet^Hi^ cells has been observed in both blood and secondary lymphoid tissues. These cells are induced by multiple stimuli, including B-cell receptor engagement, interferons, and Toll-like receptor signaling, a pattern also observed in other autoimmune diseases such as rheumatoid arthritis and SLE (6).

In patients with systemic lupus erythematosus (SLE), an increased frequency of T-bet^Hi^ B cells has been described. These cells show a substantial overlap with CD21^Low^ CD11c^+^ B-cell populations, which have also been reported to be expanded in common variable immunodeficiency (CVID). These activated B-cell subsets have been associated with disease activity, specific clinical manifestations (31, 32), and an interferon-driven transcriptional signature (33, 34).

CD21^Low^ and T-bet^+^ B cells appear to be closely related but not completely overlapping populations. T-bet has been shown to contribute to the differentiation of a subset of CD11c^+^ CD21^Low^ T-bet^+^ B cells, particularly in the context of interferon signaling and TLR stimulation (22, 35). However, not all CD21^Low^ B cells express high levels of T-bet, highlighting the heterogeneity of this compartment.

Treatment with belimumab in SLE patients has been associated with a reduction in activated B-cell populations showing partial phenotypic overlap, including T-bet^Hi^ B cells, CD11c^+^ B cells, and CD21^Low^ B cells (36), These changes likely reflect modulation of a shared activated B-cell compartment rather than independent effects on distinct subsets. In 2024, an Italian cohort of SLE patients showed an increase of these subsets which significantly decreased following belimumab administration, together with a reduction in disease activity as measured by the SLEDAI score (17, 36).

Compared to healthy donors, CVID-AI patients display increased levels of 9G4^+^ B cells, which were instead normal in CVID patients without autoimmunity. In CVID-AI patients, 9G4^+^ B cells were altered across multiple B-cell subsets: naïve B cells, transitional B cells (suggesting a defect in central or early peripheral tolerance), and several memory B-cell subsets, which in SLE have been shown to correlate with disease activity. Furthermore, increased 9G4^+^ switched memory B cells in CVID-AI support a defect in the exclusion of autoreactive B cells, a feature also observed in SLE. Similarly to SLE, CVID patients displayed increased double-negative 2 (DN2) cells (37, 38), a subset believed to represent precursors of antibody-producing plasma cells (31) and thought to be BAFF-dependent (37). Furthermore, CVID-AI patients displayed expanded populations of CD24^Hi^CD38^Hi^ B cells (39) a subset considered regulatory, as following CD40 stimulation these cells can reduce Th1 differentiation via IL-10 expression. CD24^Hi^CD38^Hi^ B cells have also been found increased in SLE; however, their IL-10 expression was impaired compared to physiological responses (40), suggesting a shared dysfunctional mechanism in both SLE and CVID-AI.

Notably, CVID-AI appears to share several key features with primary Sjögren’s syndrome (SS). Both CVID and SS are strongly associated with lymphoproliferation and an increased risk of B-cell non-Hodgkin lymphomas, including mucosa-associated lymphoid tissue (MALT) lymphomas. Elevated BAFF levels, a hallmark of CVID, are also observed in SS, which displays some of the highest BAFF concentrations among connective tissue diseases. Moreover, CD21^Low^ B cells, expanded in CVID-AI, are similarly increased in primary SS and have been linked to lymphoproliferation in that context. Finally, like SLE, primary SS and CVID-AI share a pronounced interferon signature, highlighting partially convergent pathogenetic pathways across these conditions (41).

It’s interesting to note that BAFF overexpression in murine models leads to a range of manifestations that fundamentally mirror the clinical spectrum of CVID-AI, including B-cell hyperplasia, splenomegaly, and autoimmunity, albeit with hypergammaglobulinemia, which is the opposite of what is observed in CVID patients (20). This may suggest that defects rendering TACI or BAFF-R unresponsive to BAFF somehow leads to increased BAFF levels, a hypothesis which may shed some light on CVID pathogenesis and even more in its autoimmune or inflammatory complications.

In summary, phenotypic and biological similarities between SLE, SS, and CVID-AI are numerous. Among these:

- an increase in CD21^Low^ B cells, a marker of CVID-AI (4), has also been reported in SLE, where it correlates with disease activity (27–29) and these cells are reduced following belimumab treatment (17, 36);
- elevated BAFF levels observed in CVID have been associated in murine models with splenomegaly, autoimmunity, and lymphoproliferation, and *in vitro* studies have shown that the survival of autoreactive B cells is BAFF-dependent (20).
- CVID-AI has been associated with an expansion of CD21^Low^ T-bet^Hi^ B cells (22), which are reduced by belimumab (36).

Overall, these findings suggest that BAFF-targeted therapies may modulate activated B-cell populations characterized by CD21^Low^ downregulation and T-bet expression. Whether similar mechanisms operate in CVID remains speculative, given the phenotypic and functional heterogeneity of CD21^Low^ B cells in this condition.

## Discussion

Inflammatory complications of CVID are concerning and frequent manifestations of a deep immunological dysregulation, involving different organs and systems such as the lung, liver, bowel, and bone marrow. As highlighted above, even though the main characteristic of CVID is a deficit in the B-cell compartment, activated B cells contribute to autoimmune and autoinflammatory complications. This feature explains why anti-CD20 medications such as rituximab have shown strong efficacy in achieving remission in patients with inflammatory manifestations of CVID, including cytopenias and chronic interstitial and granulomatous lung disease.

The introduction of new targeted medications is gaining increasing attention in CVID-AI, mainly proposing drugs that target the B-cell lineage, such as the proteasome inhibitor bortezomib, usually used in hematologic malignancies like multiple myeloma and capable of profoundly decreasing plasma cells, or belimumab, itself proposed as a modulator of B-cell dysregulation. Nonetheless, CVID-AI should not be reduced to a simple B-cell disease and, in fact, many therapeutic strategies have been hypothesized to target the T-cell compartment or other immunological axises, for example JAK inhibitors, abatacept, or anti-TNF agents (42).

Many of these theoretical approaches offer promising efficacy in controlling immunomediated clinical manifestations of CVID, whereas the majority of these medications carry a significant risk of adverse effects in an already compromised population such as CVID patients. Even if B-cell pathogenetic markers, whether confirmed or hypothesized to be targeted by belimumab, represent only one aspect of a shared immunopathogenic profile, the data above indicate that patients with CVID-AI, SLE, and SS exhibit overlapping serological markers and clinical features. This supports the existence of partially convergent pathogenetic pathways and suggests that similar therapeutic strategies may be effective for autoimmune or inflammatory complications of CVID.

Considering belimumab’s efficacy in SLE and SS in reducing both clinical manifestations and pathogenic cellular subsets shared with CVID-AI, and given that CVID patients already receive immunoglobulin replacement therapy, belimumab may represent a valuable therapeutic option to manage these comorbidities while reducing DMARD and steroid exposure without excessively impairing protective immune function.

To date, no clinical trials, *in vitro* studies, or case reports describing off-label belimumab use in CVID-AI have been reported. Further investigations are needed to elucidate its safety profile, *in vitro* efficacy, and clinical applicability.
